# Mechanical Properties of Titanium/Nano-Fluorapatite Parts Produced by Laser Powder Bed Fusion

**DOI:** 10.3390/ma16041502

**Published:** 2023-02-10

**Authors:** Po-Kuan Wu, Wei-Ting Lin, Jia-Wei Lin, Hong-Chuong Tran, Tsung-Yuan Kuo, Chi-Sheng Chien, Vi-Long Vo, Ru-Li Lin

**Affiliations:** 1 Department of Orthopaedics, Chi Mei Medical Center, No. 901 Zhonghua Rd., Yongkang District, Tainan 710, Taiwan; 2 Department of Mechanical Engineering, Southern Taiwan University of Science and Technology, Tainan 710, Taiwan

**Keywords:** laser powder bed fusion, biomedical implants, biocompatibility, fluorapatite material

## Abstract

Laser powder bed fusion (L-PBF) has attracted great interest in recent years due to its ability to produce intricate parts beyond the capabilities of traditional manufacturing processes. L-PBF processed biomedical implants are usually made of commercial pure titanium (CP-Ti) or its alloys. However, both alloys are naturally bio-inert, and thus reduce the formation of apatite as implants are put into the human body. Accordingly, in an attempt to improve the bioactivity of the materials used for making orthopedic implants, the present study decomposed fluorapatite material (FA, (Ca_10_(PO_4_)_6_F_2_)) into the form of nano-powder and mixed this powder with CP-Ti powder in two different ratios (99%Ti + 1%FA (Ti-1%FA) and 98%Ti + 2%FA (Ti-2%FA)) to form powder material for the L-PBF process. Experimental trials were conducted to establish the optimal processing conditions (i.e., laser power, scanning speed and hatching space) of the L-PBF process for the two powder mixtures and the original CP-Ti powder with no FA addition. The optimal parameters were then used to produce tensile test specimens in order to evaluate the mechanical properties of the different samples. The hardness of the various samples was also examined by micro-Vickers hardness tests. The tensile strength of the Ti-1%FA sample (850 MPa) was found to be far higher than that of the CP-Ti sample (513 MPa). Furthermore, the yield strength of the Ti-1%FA sample (785 MPa) was also much higher than that of the CP-Ti sample (472 MPa). However, the elongation of the Ti-1%FA sample (6.27 %) was significantly lower than that of the CP-Ti sample (16.17%). Finally, the hardness values of the Ti-1%FA and Ti-2%FA samples were around 63.8% and 109.4%, respectively, higher than that of the CP-Ti sample.

## 1. Introduction

Laser powder bed fusion (L-PBF) is one of the most common technologies in metal 3D printing manufacturing processes. In the L-PBF process, the heat energy from a controlled laser beam is used to melt the metal powder layer selectively along a specific scan path defined by the sliced geometry of the CAD file [1]. By repeating the scanning process in a layer-by-layer fashion, L-PBF enables complex geometry parts to be produced without the need for molding. It thus has strong potential for producing medical implants, which typically have a highly customized and intricate design [2,3,4,5]. However, one of the key challenges in producing such implants using the L-PBF process is that of determining the appropriate processing conditions that yield mechanical properties close to those of human bone (e.g., a Young’s modulus around 15–30 GPa) [3,4,6,7].

In an attempt to resolve this issue, several studies [2,3,8,9,10,11] have used the L-PBF process to fabricate titanium alloy lattice structures (also known as cellular or porous structures) with elastic modulus values close to that of human bone, thereby reducing the stress-shielding effect. These studies focus mainly on the effects of unit cell designs on the mechanical properties of 3D printed parts. Broadly speaking, unit cell designs can be categorized as either “scaffold-based” or “minimal surface” [7]. Chen et al. [2] produced porous Ti6Al4V scaffolds using the L-PBF process and showed that as the porosity of the porous structure increased from 43% to 71%, the Young’s modulus coefficient decreased from 55.8 to 7.8 GPa and the yield stress decreased from 565 to 62 MPa. Furthermore, for a porosity of 67%, the Young’s modulus and yield stress values (15 GPa and 129 MPa, respectively) approached those of human bone (14.8 GPa and 130 MPa, respectively), Hollander, Dirk A., et al. [8] showed that L-PBF porous structures with a pore size of 500 µm demonstrated a better bioactivity than dense structures produced by traditional manufacturing processes. Choy, Sing Ying, et al. [12] produced strut-based and honeycomb lattice structures by L-PBF processing and found that abrupt shear failure took place at the opposite corners of the lattice samples during compression testing since the diagonal struts in the planar section were oriented parallel to the compression direction. Additionally, the authors in reference [13] employed an experimental approach to find the processing conditions that can fabricate high-performance Ti6Al4V parts using the L-PBF process.

Carpenter et al. [10] were the first group to fabricate implant components with “minimal surface” designs using the L-PBF process. Several experimental studies [11,12] subsequently showed that “minimal surface” designs have numerous advantages over traditional strut-based designs, including a more efficient load transmission performance (and hence lower stress-shielding effect) than strut-based designs as a result of their greater degree of connectivity.

The long-term success of 3D printed biomedical implants depends on their biocompatibility and bioactivity. Han et al. [14] mixed nano-scale hydroxyapatite (HA) with CP-Ti and found that the resulting L-PBF processed parts had a significantly higher micro-hardness and nano-hardness than the original CP-Ti samples. Marcu et al. [15] similarly added HA to Ti6Al7Nb alloy powder to form a bio-composite powder for L-PBF processing. The experimental results showed that, given an appropriate selection of the processing conditions, the biocompatibility and mechanical properties of the printed parts were consistent with the requirements of orthopedic implant applications. Huang, Sheng, et al. [16] used the L-PBF process to produce porous titanium-tantalum scaffolds and showed that the biocompatibility of the printed samples was comparable to that of Ti6Al4V and CP-Ti samples. Aristizabal [16] fabricated orthopedic implants with excellent biocompatibility and low elastic modulus using Zr-702 alloy.

The literature contains many studies on the addition of biocompatible substances to a base material to improve the bioactivity and osseointegration of 3D printed implants. While most of these studies use HA as the doping agent, fluorapatite (FA, Ca_10_(PO_4_)_6_F_2_) also has significant potential for biomedical implants since it has the same apatite phase and a similar composition as that of human teeth and bones [17]. Furthermore, compared with HA, FA has a higher interatomic bonding strength, a lower dissolution rate, a better chemical and thermal stability, and a lower bio-resorption tendency [18,19,20]. As a result, it has many advantages over HA as a biomedical coating material, including an improved physical robustness (i.e., reduced cracking and flaking) and superior corrosion resistance. However, to the best of the current authors’ knowledge, the literature contains no studies on the addition of FA to CP-Ti powder to produce a bio-composite powder for L-PBF processing.

Accordingly, the we prepared three Ti-FA biocomposite powders with mixture ratios of 100%Ti, 99%Ti + 1%FA, and 98%Ti + 2%FA, respectively. Experimental trials were conducted to explore the optimal L-PBF processing conditions (i.e., laser power, scanning speed and hatching space) for each mixture. The mechanical properties and hardness of the specimens fabricated using the determined L-PBF parameters were then evaluated and compared.

## 2. Experimental Procedures

### 2.1. Powder Preparation

CP-Ti powder was purchased from AVIC Metal Powder Metallography Company (Taiwan). The metal powder particles were approximately spherical and had a size of 15~53 µm. In particular, the powder size distribution was analyzed by the Beckman Coulter ls320 analyzer. The particle size distribution conforms normal distribution with d10 = 19.86 µm; d50 = 31.84 µm; d90 = 49.06 µm. The chemical composition of the CP-Ti powder is shown in Table 1.

FA powder with a particle size of 0.4~0.8 μm was prepared by synthesizing Ca_3_(PO_4_)_2_ and CaF_2_ using a pressure-less stoichiometric method in accordance with the reaction 3Ca_3_(PO_4_)_2_ + CaF_2_ → Ca_10_(PO_4_)_6_F_2_ [21]. Briefly, 3Ca_3_(PO_4_)_2_ and CaF_2_ were mixed with a molar ratio of 3:1 and then further mixed with ethanol with a molar ratio of 1:3 and stirred for 24 h. The resulting mixture was dried at a temperature of 120 °C for 24 h and sieved with a standard 120 mesh. The sieved powder was placed in a furnace and heated to 300 °C at a rate of 20 °C/min. The temperature was maintained at 300 °C for 30 min and was then further increased to 500 °C at a ramp rate of 15 °C per minute. Following a heating period of 30 min, the temperature was increased to 800 °C at a ramp rate of 20 °C per minute and held for a further 30 min. Finally, the temperature was increased to 1010 °C at a heating rate of 7 °C per minute, and the powder was sintered for 9 h. Following the sintering process, the furnace was allowed to cool to room temperature and the bulk FA material was retrieved. The bulk FA was manually milled into the form of powder, and the powder was sieved with a standard 120 mesh. The filtered powder was mixed with ethanol and was then ground using aluminum grinding balls with a volume ratio of 1:3, a rotation speed of 120 rpm, and a milling time of 120 h to obtain powder particles with a size of around 1 µm. After the milling process, the FA slurry was vacuum dried to remove any excess ethanol and obtain the final desired FA product.

Bio-composite powders were prepared by adding 1 wt.% and 2 wt.% of FA powder, respectively, to CP-Ti powder. The two powder mixtures were individually mixed with ethanol in a volume ratio of 10:1. The mixtures were stirred evenly until no agglomeration was observed and were then dried in a furnace at 70 °C to remove the ethanol. Following the drying process, the powders were mixed with 20 mm alumina grinding balls in a volume ratio of 1:1 and stirred in a ball mill at 300 rpm for 5 min to obtain the desired Ti + 1%FA and Ti + 2%FA powders.

### 2.2. Laser Powder Bed Fusion Processing

The 3D-printing experiments were carried out on a commercial ITRI AM 100 machine (Industrial Technology Research Institute, Tainan, Taiwan) fitted with a fiber laser source with a wavelength of 1064 nm and a spot size of 78 µm. To prevent oxidation of the metal powder, the printing process was performed in a nitrogen environment with an oxygen concentration of less than 1000 ppm. In the L-PBF process, the quality of the top surface of each layer has a significant impact on the mechanical properties of the final component [21,22]. Accordingly, a surface-scanning technique was employed to determine the optimal processing conditions for each powder material. The processing conditions that resulted in a smooth, low-defect surface quality were then used to conduct further 3D printing trials.

### 2.3. Mechanical Properties

**Figure 1:**
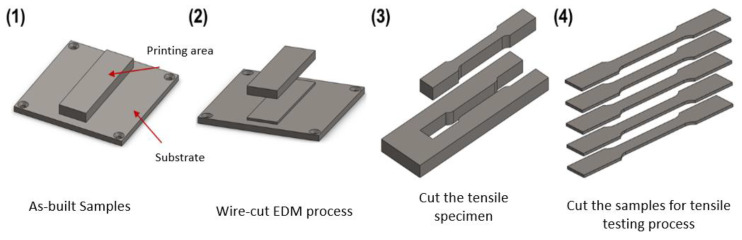
Machining of tensile test specimens.

**Figure 2:**
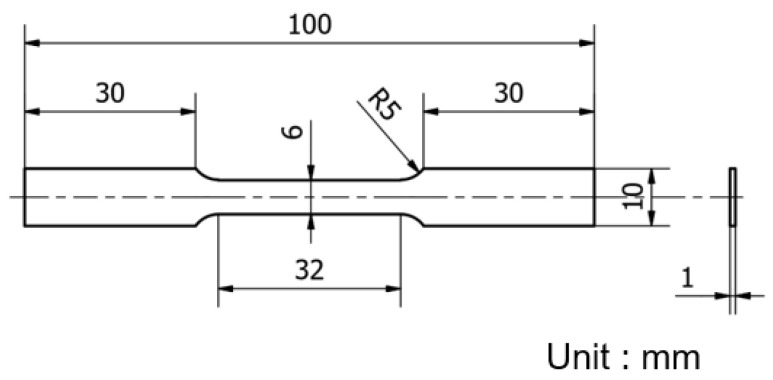
Dimensions of tensile test specimens.

The mechanical properties of the 3D printed samples were evaluated using a universal tensile testing machine (Chun Yen Testing Machines Co., Ltd., Taichung, Taiwan) with a maximum load capacity of 1000 kg. For each sample, testing was performed using an initial strain rate of 8.33 × 10^−4^ s^−1^ corresponding to a crosshead of 1 mm/min according to the standards defined in literatures [4,17]. In preparing the tensile test samples, the as-built samples machined into tensile test specimens using a wire electric discharge machining (WEDM) technique (Figure 1). The dimensions of the specimens were designed in accordance with the ASTM-E8 standard, as shown in Figure 2. The micro-hardness of the samples was measured using a Micro-Vickers hardness testing machine (HM-113, Mitutoyo, Kawasaki, Japan). The hardness tests were performed with a measurement length of 700 µm at 10× magnification and 140 µm at 50× magnification.

### 2.4. Microstructural Characterization

The microstructures and chemical compositions of the L-PBF samples were analyzed using an S-3000N scanning electron microscope (SEM, Hitachi, Tokyo, Japan) fitted with an EX-250 energy-dispersive spectroscopy (EDS) microprobe system. Phase identification was performed using a D2-PHASER X-ray diffraction (XRD) system (Bruker, Berlin, Germany) with a Cu tube and a working voltage and current of 30 kV and 10 mA, respectively. The surface roughness of the samples was analyzed using the VK-X200K confocal microscope.

## 3. Results and Discussion

### 3.1. Composite Powders

**Figure 3:**
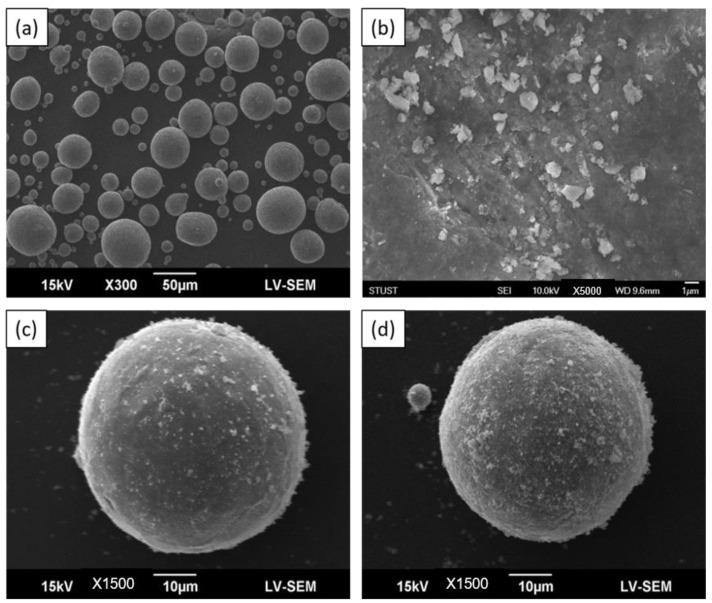
SEM images showing surface morphologies of different powder types: (**a**) CP-Ti, (**b**) FA, (**c**) Ti-1%FA, and (**d**) Ti-2%FA.

Figure 3a,b show the surface morphologies of the CP-Ti powder and FA powder, respectively. Figure 3c,d present the surface morphologies of the composite powders with 1 wt.% and 2 wt.% FA addition, respectively. For the Ti-1%FA powder, the FA particles were evenly distributed on the surface of the Ti particles, and the composite particles retained the spherical form of the original powders. For the Ti-2%FA powder, the FA particles were again uniformly distributed on the Ti particles. In general, the thermal conductivity of FA (0.02 W/mK [23]) was much lower than that of Ti (20.5 W/mK [24]). Thus, to ensure the simultaneous melting of the FA and Ti particles during the L-PBF process, the FA particles must be much smaller than the Ti particles. Accordingly, prior to mixing with the CP-Ti matrix, the FA particles were ball-milled to a size of approximately 1 µm. The small particle size increased the surface energy of the particles and hence enhanced their adhesion force to the larger CP-Ti particles. Furthermore, the use of ethanol in preparing the Ti-FA composite powders improved the uniformity of the FA particle dispersion on the Ti particle surface. During the subsequent drying process, the ethanol in the Ti-FA mixture evaporated, thereby reducing the liquid region between the FA powder and the CP-Ti particles. Consequently, a capillary force effect was induced, which further contributed to the attachment of the FA powder on the CP-Ti particle surface [25]. According to [25], spherical metal powders increase the flow-ability of the metal powder layer in the L-PBF process, and hence improve the stability of the printing process. The SEM images in Figure 3c,d confirm that both composite powders have a spherical morphology and are thus suitable as a feedstock material for L-PBF processing.

**Figure 4:**
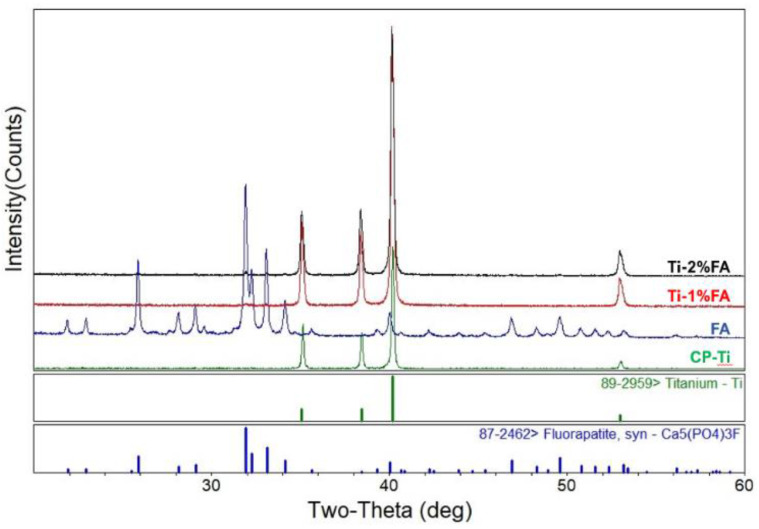
XRD patterns of different powder types.

**Figure 5:**
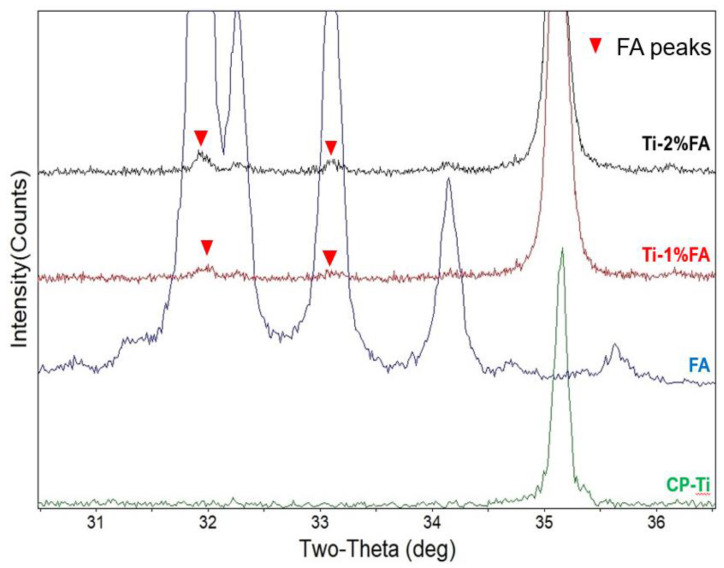
XRD patterns of different powder types in main peak range of FA.

Figure 4 shows the XRD pattern of the composite powders; additionally, for comparison purposes, this figure also shows the XRD patterns of the original FA and CP-Ti powders. However, it is hard to observe the FA peaks in composite powders. For clearly demonstrating the existence of FA peak in composite powders, Figure 5 is presented. From Figure 5, it is seen that for the composite powders, the FA peak increases with an increasing FA addition. Nevertheless, since the maximum FA addition is just 2%, the FA peak is still very weak compared with that of the Ti peak in the composite samples.

In summary, from the SEM images shown in Figure 3c,d, it is observed that the FA nanoparticles disperse uniformly on the single CP-Ti powders. Furthermore, the XRD patterns shown in Figure 4 and Figure 5 further confirm the present of FA peak in the composite powders. Thus, it can be verified that by using the proposed approach, the FA nanoparticles are able to spread uniformly on the surface of CP-Ti particles.

### 3.2. Determination of Optimal Processing Parameters for L-PBF Experiments

The L-PBF process fabricates 3D parts in a layer-by-layer manner [22]. Consequently, the quality of the top surface of the intermediate layers has a significant effect on the mechanical properties of the final component. Accordingly, a series of trials was performed to determine the optimal processing conditions (i.e., laser power, scanning speed, and hatching space) for the present CP-Ti, Ti-1%FA. and Ti-2%FA powders. For each material, cubic samples with a size of 10 mm × 10 mm × 0.4 mm were printed using a constant laser power of 150 W and the scanning speed and hatching space settings shown in Table 2. The surface morphologies of the top surfaces of the cubic samples were then analyzed by SEM to determine the optimal processing conditions for each of the powder materials.

**Figure 6:**
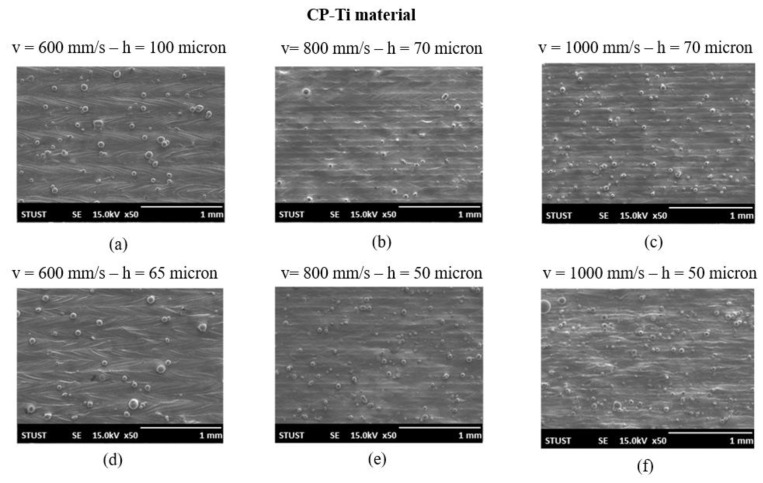
SEM surface images of as-built CP-Ti samples fabricated using different scanning speeds and hatching spaces: (**a**) v = 600 mm/s—h = 100 µm; (**b**) v = 800 mm/s—h = 70 µm; (**c**) v = 1000 mm/s—h = 70 µm; (**d**) v = 600 mm/s—h = 65 µm; (**e**) v = 800 mm/s—h = 50 µm; (**f**) v = 1000 mm/s—h = 50 µm.

Figure 6 presents SEM images of the top surface morphologies of the six CP-Ti samples fabricated using the various processing conditions listed in Table 2. It is seen that for the lowest scanning speed of 600 mm/s (Figure 6a,d), the high energy density led to extreme evaporation during the printing process, and hence the sample surface contained a large number of spattered particles. As the scanning speed increased to 800 mm/s, the energy density reduced, and thus the sample produced with a hatching space of 70 µm had a smooth surface (Figure 6b). However, for a lower hatching space of 50 µm, the surface contained a large number of tiny particles (Figure 6e) since the narrow hatching space increases the energy input density and therefore intensifies the evaporation effect. The resulting small spatters were not transported away from the melting zone by the air flow in the chamber, and hence settled back on the sample surface in the form of small particles [26]. As the scanning speed was further increased to 1000 mm/s, the melt pool became unstable and gave rise to a poor surface roughness [21,27]. Consequently, for both settings of the hatching space, the sample surface contained many tiny particles (Figure 6c,f). In general, the results presented in Figure 4 indicate that the optimal L-PBF processing conditions for the CP-Ti samples were a laser power of 150 W, a scanning speed of 800 mm/s, and a hatching space of 70 µm.

**Figure 7:**
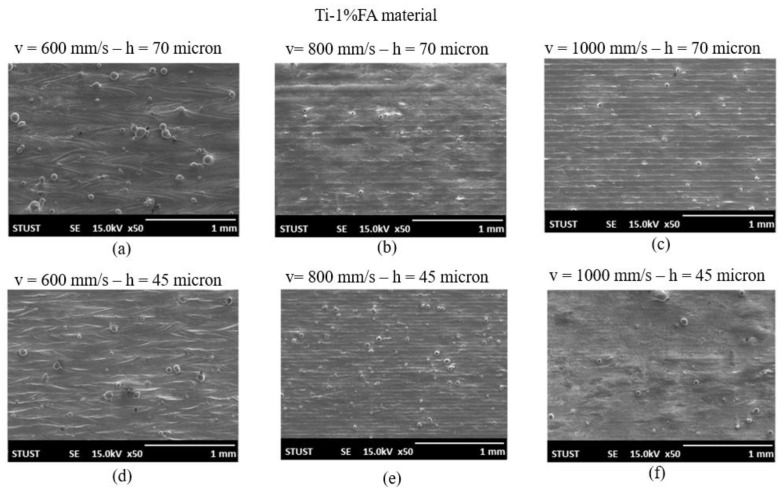
SEM surface images of as-built Ti-1%FA samples fabricated using different scanning speeds and hatching spaces: (**a**) v = 600 mm/s—h = 70 µm; (**b**) v = 800 mm/s—h = 70 µm; (**c**) v = 1000 mm/s—h = 70 µm; (**d**) v = 600 mm/s—h = 45 µm; (**e**) v = 800 mm/s—h = 45 µm; (**f**) v = 1000 mm/s—h = 45 µm.

Figure 7 shows SEM images of the Ti-1%FA samples fabricated using the processing conditions listed in Table 2. For a low scanning speed of 600 mm/s, the sample surface contained a small number of spattered particles due to the intense evaporation effect induced by the high energy density input and the ejection of molten material from the melt pool (Figure 7a,d). For a scanning speed of 800 mm/s, the sample surface had a smooth appearance, particularly in the case of a wider hatching space (Figure 7b,e). The number of spattered particles reduced yet further as the scanning speed increased to 1000 mm/s (Figure 7c,f). Interestingly, a comparison of Figure 6 and Figure 7 shows that, for same laser power of 150 W, scanning speed of 800 mm/s, and nearly the same hatching space, the Ti-1%FA sample had fewer spatters on the sample surface than the pure CP-Ti sample. This result is reasonable since the addition of FA to the titanium matrix increases the viscosity of the melt pool [28]. Consequently, the ejection of molten particles from the melt pool under the same laser energy density was suppressed, and hence the surface roughness of the sample was improved. Overall, the results presented in Figure 7 show that for the Ti-1%FA powder, the optimal processing conditions were a laser power of P = 150 W, a scanning speed of 1000 mm/s, and a hatching space of 70 µm.

**Figure 8:**
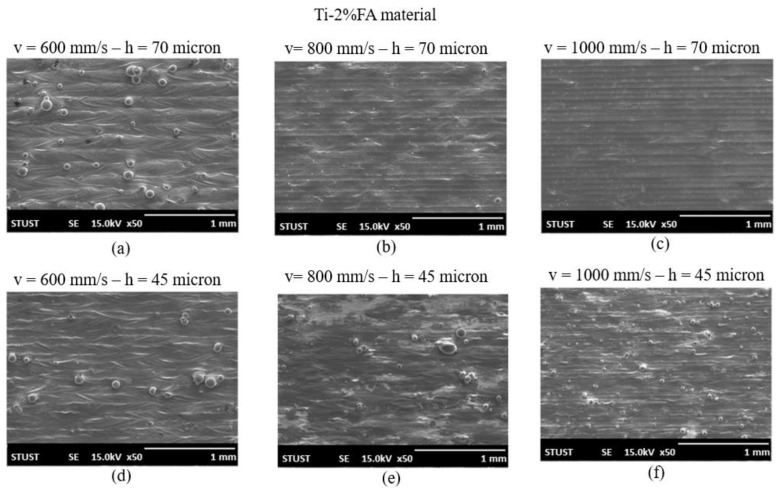
SEM surface images of as-built Ti-2%FA samples fabricated using different scanning speeds and hatching spaces: (**a**) v = 600 mm/s—h = 70 µm; (**b**) v = 800 mm/s—h = 70 µm; (**c**) v = 1000 mm/s—h = 70 µm; (**d**) v = 600 mm/s—h = 45 µm; (**e**) v = 800 mm/s—h = 45 µm; (**f**) v = 1000 mm/s—h = 45 µm.

Figure 8 presents SEM images of the Ti-2%FA samples fabricated using the processing conditions listed in Table 2. As for the as-built Ti-1%FA samples, the optimal processing parameters were seen to be a laser power of 150 W, a scanning speed of 1000 mm/s, and a hatching space of 70 µm.

### 3.3. Microstructure Analysis of L-PBF Parts Fabricated Using Optimal Processing Conditions

**Figure 9:**
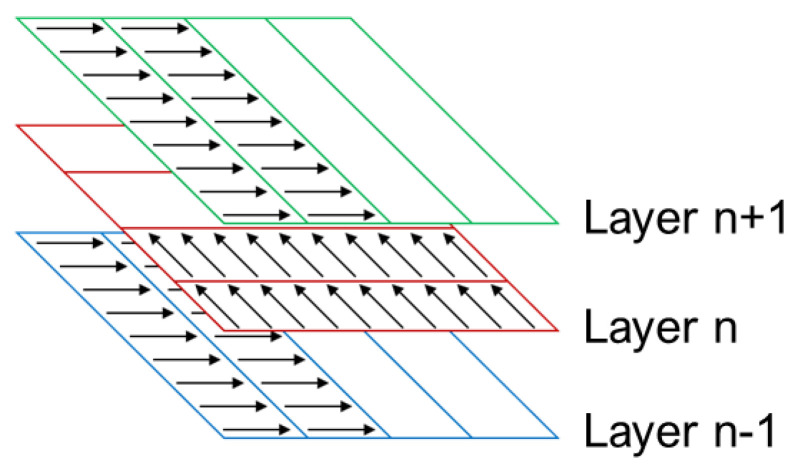
Stripe scanning strategy employed in the L-PBF process.

The optimality of the processing conditions determined in the previous section (summarized in Table 3) was confirmed by performing further L-PBF printing trials using a larger cubic sample size of 80 mm × 40 mm × 10 mm. In performing the printing process, the rotation angle between adjacent layers was set as 90° in order to reduce the accumulation of residual stress. Moreover, a stripe scan strategy was employed with a stripe width of 5 mm (see Figure 9). The relative density of the printed components was evaluated using an optical microscopy (OM) technique [29,30].

**Figure 10:**
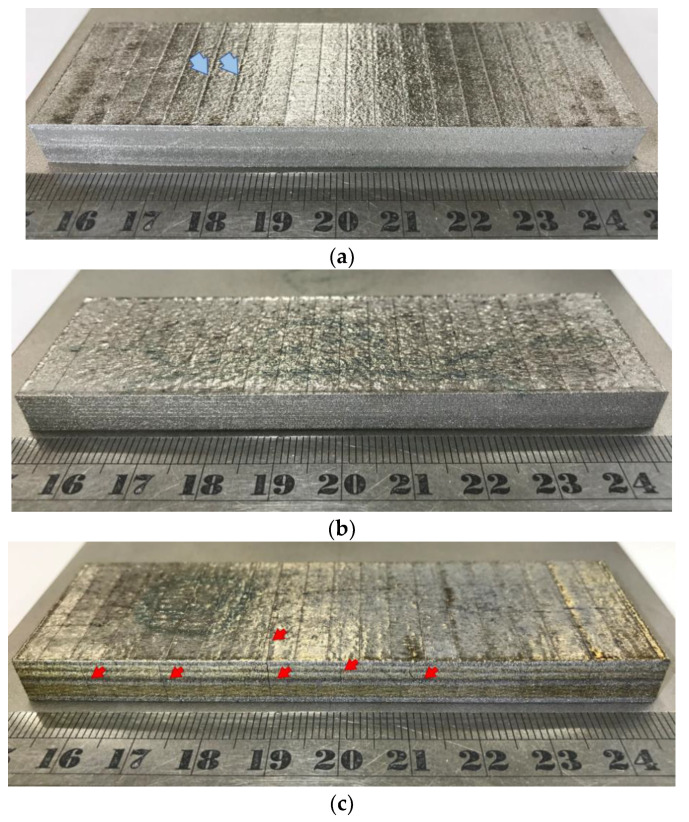
L-PBF samples processed using optimal parameters: (**a**) CP-Ti, (**b**) Ti-1%FA, and (**c**) Ti-2% FA.

Figure 10a–c show the 3D printed samples fabricated using CP-Ti, Ti-1%FA, and Ti-2%FA powders, respectively. The top surface of the CP-Ti sample and Ti-1%FA contains distinct parallel lines originating from the use of the stripe scanning strategy (Figure 10a,b). As shown in Figure 10c, the Ti-2%FA sample contains several cracks on the side surface running parallel to the built-up direction. During the L-PBF process, the cooling rates and thermal gradients around the melt pool are very high and hence produce significant stress. If the accumulation of stress exceeds the yield stress of the material, the built part experiences permanent deformation, and may crack. Furthermore, during the L-PBF process, the FA particles undergo thermal decomposition, and the resulting components may then react with CP-Ti to form low-ductility brittle phases such as Ti_x_P_y_ [14]. The combination of a high stress and low ductility then leads to the occurrence of cold cracks, as shown in Figure 10c.

**Figure 11:**
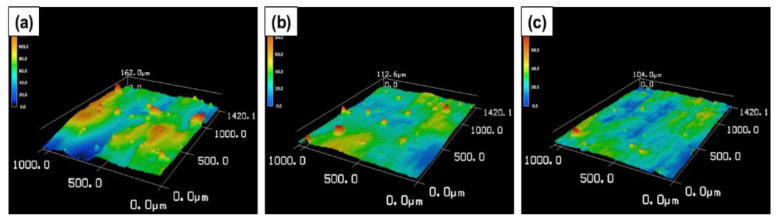
As-built surface morphology of (**a**) CP-Ti, (**b**) Ti-1%FA, (**c**) Ti-2%FA.

**Figure 12:**
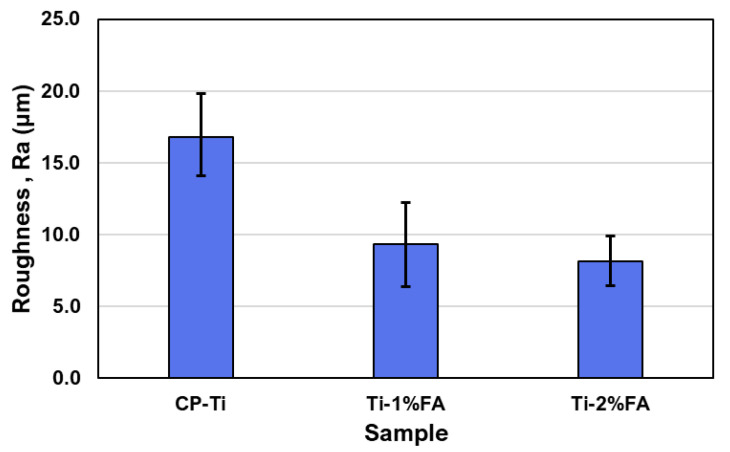
Comparisons between surface roughness of as-built samples of three different material.

Figure 11 and Figure 12 show the surface morphology and surface roughness analysis of CP-Ti, Ti-1%FA and Ti-2%FA L-PBF processed samples. It is seen that the measured roughness (Ra) of CP-Ti specimens was higher (about 16.8 μm) among the three materials, while that of Ti-1%FA (about 9.3 μm) and Ti-2%FA (about 8.1 μm) specimen surface was lower.

**Figure 13:**
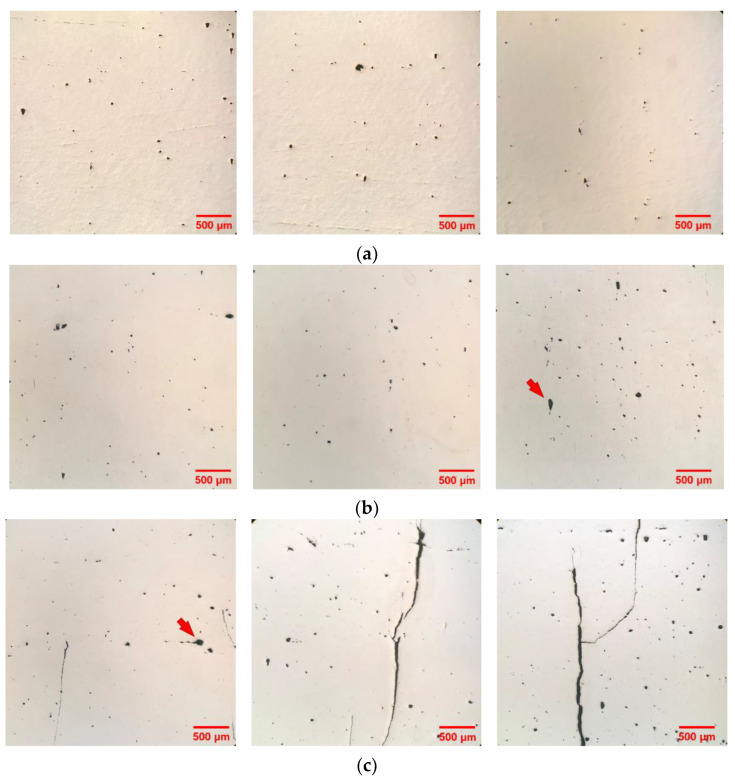
OM images showing cross-sections of various as-built samples: (**a**) CP-Ti, (**b**) Ti-1%FA, (**c**) Ti-2%FA.

Figure 13a–c present typical OM cross-sectional images of the CP-Ti, Ti-1%FA, and Ti-2%FA as-built samples, respectively. It was seen that even though all three samples were fabricated using the optimal processing conditions (as determined by surface scanning experiments), the cross-sections still contained pores, voids, and (in some cases) cracks. It was reported in [31] that the origins of the pores formed in the L-PBF process can be classified into three main types: (1) poor-adhesion, (2) key-hole, and (3) gas bubble entrapment during the solidification process [32,33]. It was additionally noted that the pores formed by gas bubble entrapment have a characteristic size of around 10 µm. A careful inspection of the OM images in Figure 13a suggests that the pores within the as-built CP-Ti sample may thus belong to the third type. This assertion is reasonable since the CP-Ti powder has a high oxygen concentration of around 0.13 wt.% (see Table 1). Part of this oxygen escapes from the melt pool as a result of the melt pool flow dynamics. However, the remainder remains trapped in the melt pool during the solidification process and produces small pores within the final CP-Ti component as a result.

An observation of Figure 13b shows that the Ti-1%FA samples also contain relatively large pores, albeit in a slightly lower quantity than the CP-Ti samples. The size of the pores again suggests that they are the result of gas bubble inclusion (oxygen or nitrogen) during the solidification process. The Ti-2%FA samples (Figure 13c) showed the appearance of many cold cracks as a result of a high residual stress accumulation and a low ductility of the composite material. It is also suspected that during the L-PBF process, cracking debris detached from the substrate and interfered with the powder re-coater. Thus, the spreading uniformity of the powder particles on the powder bed surface was impaired. Consequently, the cross-section of the Ti-2%FA sample contained not only cold cracks, but also a large number of pores.

**Figure 14:**
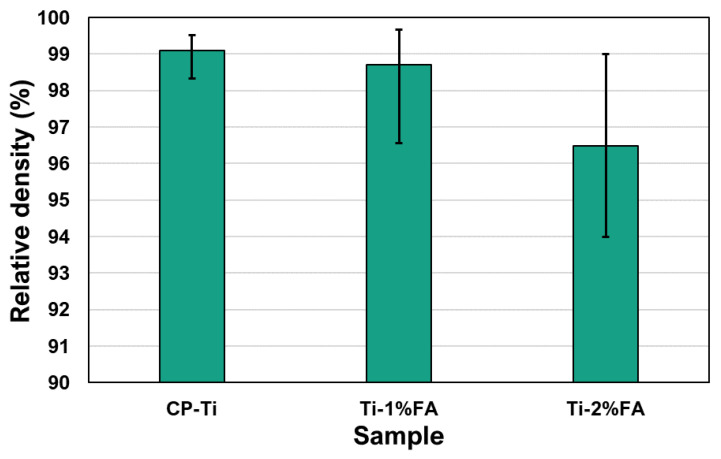
Relative densities of as-built samples.

The relative densities of the three samples were measured using the method described in [29]. The corresponding results are presented in Figure 14. Among the three samples, the CP-Ti sample had the highest relatively density of 99.1%. The relatively density reduced slightly to 98.7% for the sample containing 1 wt.% FA, and then reduced more significantly to 96.5% as the FA addition was further increased to 2 wt.%. The weight percentage of FA in Ti-2%FA composite powders was higher than that of Ti-1%FA composite powders. During L-PBF process, the FA particles underwent thermal decomposition, and the resulting components may then reacted with CP-Ti to form low-ductility brittle phases such as Ti_x_P_y_ [4]. Thus, the number of brittle phases in as-built Ti-2%FA is expected to be higher than that of Ti-1% FA. The brittle phases together with the accumulation of tensile residual stress will lead to the cold crack along the build direction [34] (shown in Figure 13c). These cold cracks will degrade the density of as-built Ti-2%FA samples. Therefore, the density of Ti-2%FA samples was lower than that of Ti-1%FA samples.

### 3.4. XRD and EDS Analysis

**Figure 15:**
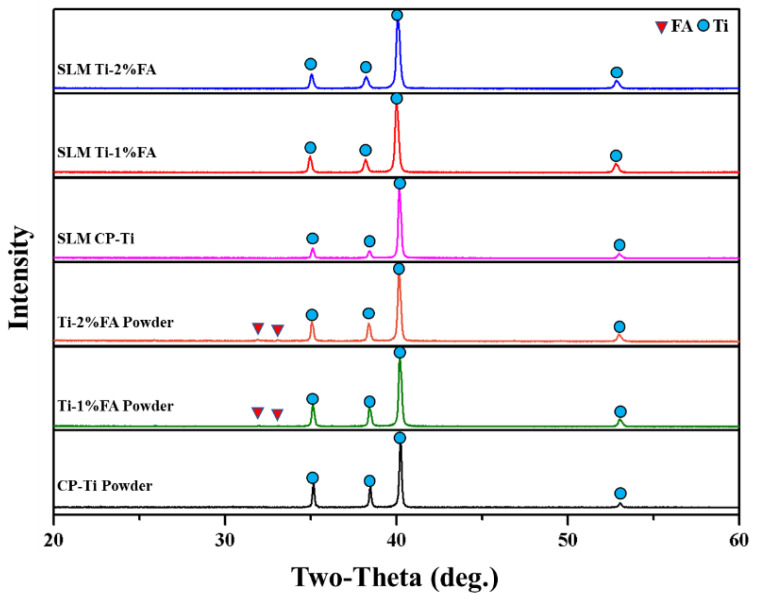
XRD patterns of L-PBF powders and as-built samples.

Figure 15 shows the XRD patterns of the three powder mixtures and corresponding as-built parts. A strong peak corresponding to FA was observed for both composite powders. However, the patterns for the corresponding as-built parts showed no FA diffraction peaks and contained only Ti peaks. The absence of FA peaks is due in part to the low quantity of FA powder added to the Ti matrix (1~2 wt.%) Furthermore, during the L-PBF process, the FA undergoes thermal decomposition, and some of the FA reacted with Ti to form Ti_x_P_y_, CaTiO_3_, or Ti_x_O [28]. Consequently, the quantity of FA within the Ti matrix was further reduced. Notably, the authors in [14] reported a similar absence of HA peaks in the XRD patterns of as-built Ti-HA samples.

**Figure 16:**
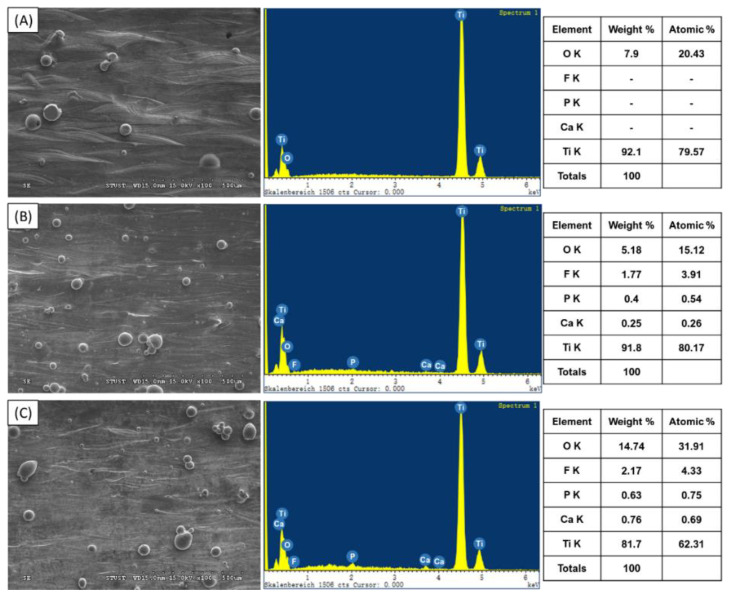
EDS analysis results for as-built samples: (**A**) CP-Ti, (**B**) Ti-1%FA, and (**C**) Ti-2%FA.

The FA-related contents of the three samples were further investigated by EDS (Figure 16). The results indicated that, despite the absence of FA peaks in the XRD patterns, the Ti-1%FA and Ti-2%FA samples did in fact contain a small quantity of FA-related substances (Ca, P, F). Furthermore, as expected, the quantity of these substances increased slightly with an increasing FA.

### 3.5. Hardness and Tensile Properties

**Figure 17:**
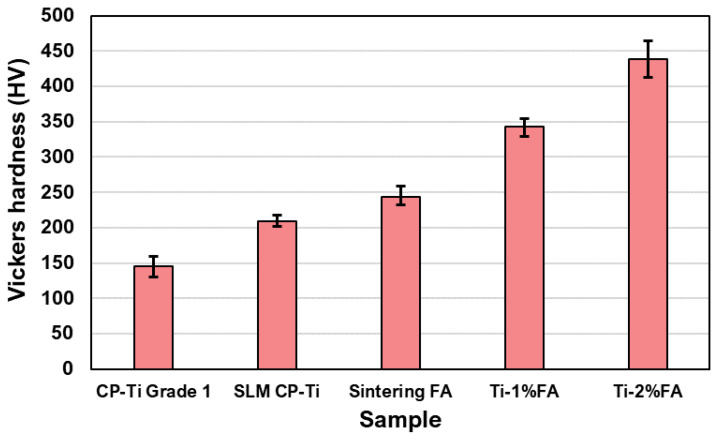
Measured hardness values of different samples.

Figure 17 shows the hardness values of the as-built CP-Ti, Ti-1%FA and Ti-2%FA samples. For comparison purposes, the figure also shows the hardness of an as-cast CP-Ti Grade 1 sample [35] and a sintered FA sample. The as-cast CP-Ti sample had a hardness of around 150 HV. By contrast, the hardness of the present as-built CP-Ti sample was around 209 HV, which is about 44.1% higher than that of the as-cast sample. The higher hardness of the present sample can be attributed to the rapid heating and cooling characteristics of the laser heating source. In particular, a large number of thermal cycles were generated in the L-PBF process during the repeated scanning operations. Consequently, the residual stress (and hence the hardness) of the as-built part increased [29]. The sintered FA sample had a hardness of 244 HV, which is around 16.7% higher than that of the as-built CP-Ti sample. For the sample with 1 wt.% FA addition, the hardness increased to 342.4 HV, i.e., 63.8% higher than that of the CP-Ti sample and 40% higher than that of the sintered FA sample. Finally, the Ti-2%FA sample had a high hardness of 438.6 HV, i.e., about 63.4% higher than that of the Ti-1%FA sample and 109.4% higher than that of the as-built CP-Ti sample. In general, the results showed that the hardness of the samples increased with an increasing FA content. It is speculated that the hardness improvement is the result of FA thermal decomposition during the L-PBF process and the subsequent formation of hard and brittle phases in the as-built sample [14].

As described above, the Ti-2%FA sample showed significant cold cracks (Figure 13c). Therefore, its tensile strength is expected to be significantly lower than the CP-Ti. It was thus excluded from the tensile test experiments. CP-Ti and Ti-1%FA samples were prepared using the conditions shown in Table 3.

**Figure 18:**
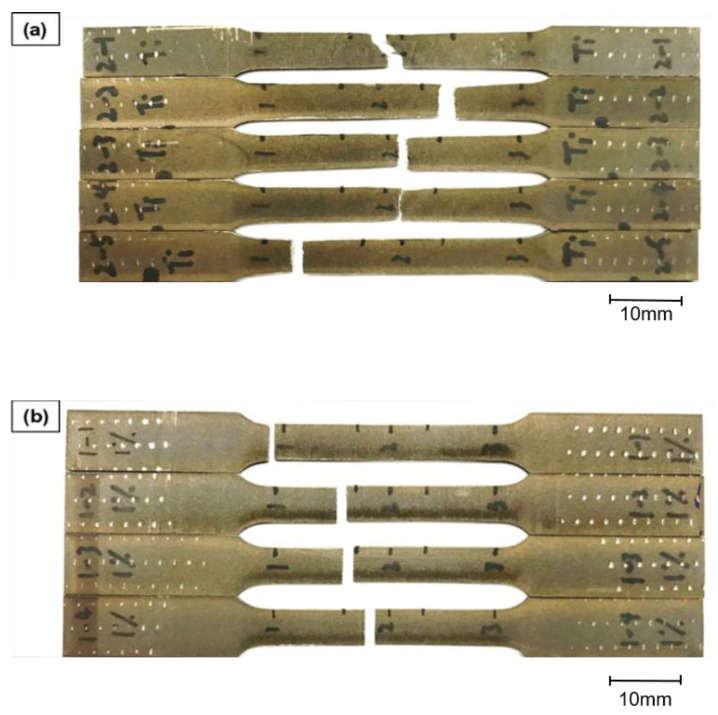
Fracture samples after tensile tests: (**a**) Cp-Ti, and (**b**) Ti-1%FA.

Figure 18a,b show the CP-Ti and Ti-1%FA fractured samples, respectively after the tensile tests. As shown in Figure 18a, the CP-Ti specimens all demonstrated a necking phenomenon followed by shear failure. By contrast, the Ti-1%FA samples exhibited no necking zone and had a brittle fracture morphology at the fracture point. Hence, it is inferred that, of the two samples, the CP-Ti samples have a higher toughness.

**Figure 19:**
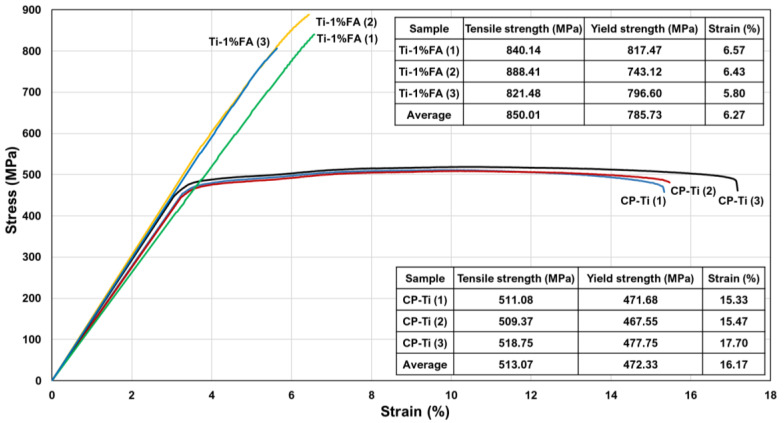
Stress vs. strain response of CP-Ti and Ti-1%FA samples.

**Figure 20:**
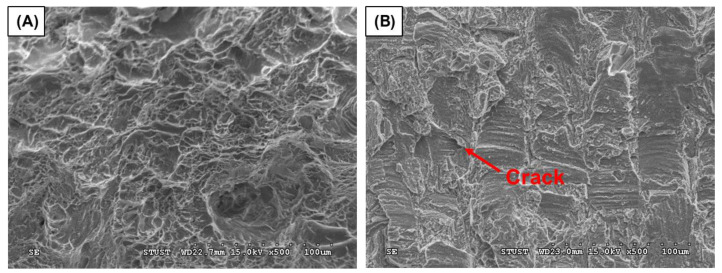
SEM images showing cross-section fracture surfaces of tensile specimens: (**A**) CP-Ti, and (**B**) Ti-1%FA.

Figure 19 presents the tensile test results for the CP-Ti and Ti-1%FA samples. It was seen that the Ti-1%FA sample has an average tensile strength of 850 MPa, which is around 65% higher than that of the CP-Ti sample (513 MPa). Moreover, the yield strength of the Ti-1%FA sample (785 MPa) was around 84% higher than that of the CP-Ti sample (427 MPa). However, the elongation of the Ti-1%FA sample (6.27%) was around 61% lower than that of the CP-Ti sample (16.17%). In other words, the addition of 1 wt.% FA to the Ti matrix increased the strength of the as-built sample, but reduced the ductility. This can be explained by the fact that the addition of 1 wt.% to the CP-Ti matrix may lead to the formation of Ti_x_P_y_, CaTiO_3_ due to the thermal decomposition of FA (Ca_10_(PO_4_)_6_F_2_)) [28]. These two phases make the composite material become brittle. As a result, no obvious necking behavior was observed at the fracture location. Therefore, the elongation of Ti-1%FA is significantly lower than that of CP-Ti. The SEM image presented in Figure 20A shows that the fracture surface of the CP-Ti sample contains dimple-like features characteristic of a ductile (i.e., high toughness) material. By contrast, the fracture surface of the Ti-1%FA sample showed cleavage facets, a small number of shallow dimples, and several cracks, which indicate a brittle nature of the sample (Figure 20B).

Han et al. [14] added HA to CP-Ti powder to form a bio-composite powder and found that the HA decomposed during the L-PBF process to form Ti_x_P_y_ and CaTiO_3_ phases. These phases increased the brittleness of the as-built samples. Accordingly, the tensile specimens showed no obvious necking behavior prior to fracture. In this work, as described above, the addition of 1 wt.% FA to the CP-Ti matrix reduced the elongation of the as-built Ti-1%FA sample, but increased the tensile strength. This can be explained by the fact that the thermal decomposition temperature of FA (1400 °C) is much higher than that of HA (800–850 °C). Thus, the number of brittle phases formed in the CP-Ti matrix during the L-PBF process was lower than that produced in the Ti-HA sample. Overall, the results suggest the desirability for utilizing FA in place of HA to minimize the loss of strength caused by thermal decomposition during the L-PBF process.

## 4. Conclusions

This study developed a pressure-less stoichiometric technique to prepare composite Ti-1%FA and Ti-2%FA powders for L-PBF processing. The feasibility of the proposed approach was confirmed by the SEM and XRD analysis results. In particular, the SEM observations showed that the FA nanoparticles were uniformly distributed on the surface of the Ti powder. In addition, the XRD results revealed strong FA-related peaks in the XRD pattern of the composite powder. The composite powders were used to fabricate 3D L-PBF parts under optimal processing conditions. It was shown that the Ti-1%FA and Ti-2%FA specimens had relative densities of 98.5% and 96.5%, respectively, and are thus more porous than a pure CP-Ti sample with no FA addition (99%). The lower relative density of the Ti-2%FA sample is attributed to the formation of brittle phases during thermal decomposition and the accumulation of a high residual stress during the L-PBF process, which collectively led to the formation of a large number of cracks on the top and side surfaces of the as-built sample.

The micro-Vickers hardness tests showed that the CP-Ti sample hardness (209 HV) was around 44.1% higher than that of a traditional cast CP-Ti sample 150 HV). Furthermore, the hardness values of the Ti-1%FA (342.4 HV) and Ti-2%FA (438.6 HV) samples were around 63.8% and 109.4%, respectively, higher than that of the printed CP-Ti sample. The higher hardness of the Ti-1%FA and Ti-2%FA samples is most likely the result of the thermal decomposition of the FA during the L-PBF process to form hard and brittle phases such as Ti_x_P_y_. Finally, the tensile test results showed that the tensile strength and yield strength of the Ti-1%FA sample were around 65% and 66%, respectively, higher than those of the CP-Ti sample with no FA addition. However, the elongation was reduced by around 61%. The higher hardness but lower toughness of the Ti-1%FA sample is attributed to the formation of brittle phases during the L-PBF process.

For future works, thermos-mechanical simulation will be developed to predict the parameters that lead to lower residual stress. These parameters will be utilized to manufacture Ti-2%FA for preventing crack. Additionally, the Ti-1%FA will be further employed for 3D printing lattice structures with elastic modulus close to that of human bone for reducing the stress-shielding effects [6].

## Figures and Tables

**Table 1:** Chemical composition of the CP-Ti powder.

Element	N	H	O	C	Fe	Ti
Wt%	0.04 max	0.0125 max	0.13 max	0.08 max	0.2 max	Bal.

**Table 2:** Processing conditions for surface scanning experiments.

Fixed Processing Conditions for All Materials		
Powder size (µm)		<55
Diameter of laser beam (µm)		78
Layer thickness (µm)		40
Oxygen concentration (ppm)		O_2_ < 1000 ppm
Laser power (W)		150
Parameter Settings for Different Materials		
CP-Ti	Scanning Speed (mm/s)	Hatching Space (µm)
	600	100
		65
	800	70
		50
	1000	70
		50
Ti-1%FA Ti-2%FA	600	70
		45
	800	60
		40
	1000	60
		40

**Table 3:** Processing conditions for 3D-printing experiments.

Fixed Processing Conditions for All Materials		
Powder size (µm)		<55
Diameter of laser beam (µm)		78
Layer thickness (µm)		40
Oxygen concentration (ppm)		O_2_ < 1000 ppm
Laser power (W)		150
Parameter Settings for Different Materials		
CP-Ti	Scanning Speed (mm/s)	Hatching Space (µm)
	800	70
Ti-1%FA	1000	70
Ti-2%FA	1000	70

## Data Availability

Not applicable.
